# Hemodiafiltration is associated with reduced inflammation, oxidative stress and improved endothelial risk profile compared to high-flux hemodialysis in children

**DOI:** 10.1371/journal.pone.0198320

**Published:** 2018-06-18

**Authors:** Ayşe Ağbaş, Nur Canpolat, Salim Çalışkan, Alev Yılmaz, Hakan Ekmekçi, Mark Mayes, Helen Aitkenhead, Franz Schaefer, Lale Sever, Rukshana Shroff

**Affiliations:** 1 Department of Pediatric Nephrology, Istanbul University Cerrahpasa Faculty of Medicine, Istanbul, Turkey; 2 Department of Pediatric Nephrology, Istanbul University Istanbul Faculty of Medicine, Istanbul, Turkey; 3 Department of Biochemistry, Istanbul University Cerrahpasa Faculty of Medicine, Istanbul, Turkey; 4 Department of Pediatric Nephrology, Great Ormond Street Hospital for Children NHS Foundation Trust, London, United Kingdom; 5 Department of Chemical Pathology, Great Ormond Street Hospital for Children NHS Foundation Trust, London, United Kingdom; 6 Department of Pediatric Nephrology, Center for Pediatrics and Adolescent Medicine, Heidelberg University Hospital, Heidelberg, Germany; University of Milan, ITALY

## Abstract

Randomized trials in adults have shown reduced all-cause and cardiovascular mortality on hemodiafiltration (HDF) compared to high-flux hemodialysis (HD), but the mechanisms leading to improved outcomes are not clear. We studied biomarkers of inflammation, oxidative stress, anti-oxidant capacity and endothelial dysfunction in 22 children (13 female, age 8–15 years). All children received HD for at least 3 months, and were then switched to HDF, keeping all dialysis related parameters and dialysis time constant. All the biomarkers of inflammation (ß2-microglobulin, IL-6, IL-10, high sensitive C-reactive protein [hsCRP]), oxidative stress (nitrotyrosine, advanced glycation end-products [AGEs], oxidized low density lipoprotein [ox-LDL] and anti-oxidant capacity) and endothelial dysfunction (asymmetric dimethyl arginine [ADMA], symmetric dimethyl arginine [SDMA]), were comparable between incident and prevalent patients on HD, suggesting that even a short dialysis vintage of 3 months on HD increases inflammation and endothelial stress. After 3 months of HDF therapy there was a significant reduction in ß2-microglobulin (p<0.001), hCRP, ADMA, SDMA, AGEs, ox-LDL (p<0.01 for all) and an increase in total antioxidant capacity (p<0.001) compared to HD. All children were maintained on the same dialyser, dialysis water quality, dialysis time and blood flow speeds suggesting that improved clearances on HDF led to an improved biomarker profile. Even in children with residual renal function there was a significant reduction in ß2 microglobulin, hsCRP, SDMA, ox-LDL and AGEs on HDF compared to HD. Children with a lower blood flow had higher inflammatory status (higher IL-6/IL-10 ratio; p = 0.04, r = -0.43). Children who achieved a higher convective volume (≥median 12.8L/m^2^) had lower ox-LDL (p = 0.02). In conclusion, we have shown that a significant improvement in inflammation, antioxidant capacity and endothelial risk profile is achieved even within a short time (3 months) on HDF compared to HD treatment.

***Trial Registration*:** ClinicalTrials.gov: NCT02063776.

## Introduction

The survival of children with end stage kidney disease (ESKD) has increased over the years, but their mortality remains significantly higher than healthy peers, with cardiovascular disease accounting for 30% of deaths on dialysis [1,2]. Non-traditional risk factors for cardiovascular disease such as increased oxidative stress, inflammation and endothelial dysfunction [3,4] are commonly present in ESKD, and are causally associated with intimal disease or atherosclerosis [1,5]. Uremic toxins such as advanced gylcation end products (AGEs) and asymmetric dimethylarginine (ADMA) are thought to play a direct role in the pathogenesis of the endothelial dysfunction [6,7], while others such as β2-microglobulin (β2M) is associated with increased mortality [8].

Hemodiafiltration (HDF) is a newer technique of dialysis that achieves clearance of middle and large molecular weight solutes unlike conventional hemodialysis (HD). HD is based on the diffusive transport of solutes across a semipermeable membrane and is effective in removing small solutes only, whereas HDF also involves the infusion of sterile, pyrogen-free fluid either pre- or post-filter and thereby allows clearance by convection as well as diffusion [9,10]. Analysis of pooled individual participant data from randomized controlled trials has shown survival benefits of high volume-HDF on all-cause mortality and especially cardiovascular mortality rate [11–13]. The mechanisms that lead to improved outcomes are not clear, but it is thought that HDF may reduce the production of inflammatory mediators through the use of biocompatible dialysers and ultra-pure dialysate and also improve clearance of larger molecular weight substances, many of which are associated with oxidative stress, inflammation and endothelial dysfunction [14,15]. However it is not clear if this improved removal of inflammatory mediators translates into better clinical outcomes [9].

HDF is increasingly used in adults and children, but there is little data on its benefits, if any, in children [16]. Fischbach et al showed improved nutrition and growth and low serum C-reactive protein (CRP) levels in 15 children undergoing daily HDF [17]. A further study has demonstrated reduced CRP and interleukin-6 (IL-6) in children on intermittent thrice-weekly HDF [18,19], with improved cardiac systolic function in a sub-group [18]. However, some of these studies compared low-flux HD with HDF, did not standardize dialysis parameters on HD and HDF to allow a true comparison of the effectiveness of each dialysis modality, and did not describe outcomes relative to the achieved convective volume on HDF.

We studied 22 children on chronic HD for at least 3 months and then switched them to HDF, standardizing all other dialysis related parameters and dialysis time, in order to determine the intra-individual changes in markers of oxidative stress (nitrotyrosine, AGEs, oxidized low density lipoprotein [ox-LDL], total antioxidant capacity [TAC]), inflammation (ß2M, IL-6, IL-10, high sensitive C-reactive protein [hsCRP]), and endothelial dysfunction (ADMA, symmetric dimethyl arginine [SDMA]), on different dialysis modalities.

## Materials and methods

### Study population

We performed a prospective observational study in 22 children on chronic HD who were dialysed in two tertiary nephrology units between 2013 and 2016. This study is a sub-study of the larger ‘Hemodiafiltration, Heart and Height (3H) study’ and is registered under ClinicalTrials.gov: NCT02063776. The authors confirm that all ongoing and related trials for this study are registered. The trial protocol is available; S1 protocol.

Children above 5 years of age without underlying chronic inflammatory or autoimmune diseases, diabetes or immune deficiencies, without active infections in the preceding 3 months, and non-smokers were included. Thirteen consecutive children who were starting HD from Great Ormond Street Hospital for Children, London and nine consecutive prevalent HD patients from Istanbul University who fulfilled the inclusion criteria were recruited. The median dialysis vintage in prevalent HD patients was 36 (range 3.5 to 58) months. Underlying kidney diseases were congenital anomalies of kidney and urinary tract (n = 11), glomerular disease (n = 3), neurogenic bladder (n = 3), cystinosis (n = 2), unknown (n = 3). The study was approved by research ethics committees in London and Istanbul. Written informed consent was obtained from all parents or guardians and assent was obtained from children where appropriate. The study was conducted according to the guideline of Good Clinical Practice and the principles of the Declaration of Helsinki.

**Fig 1:**
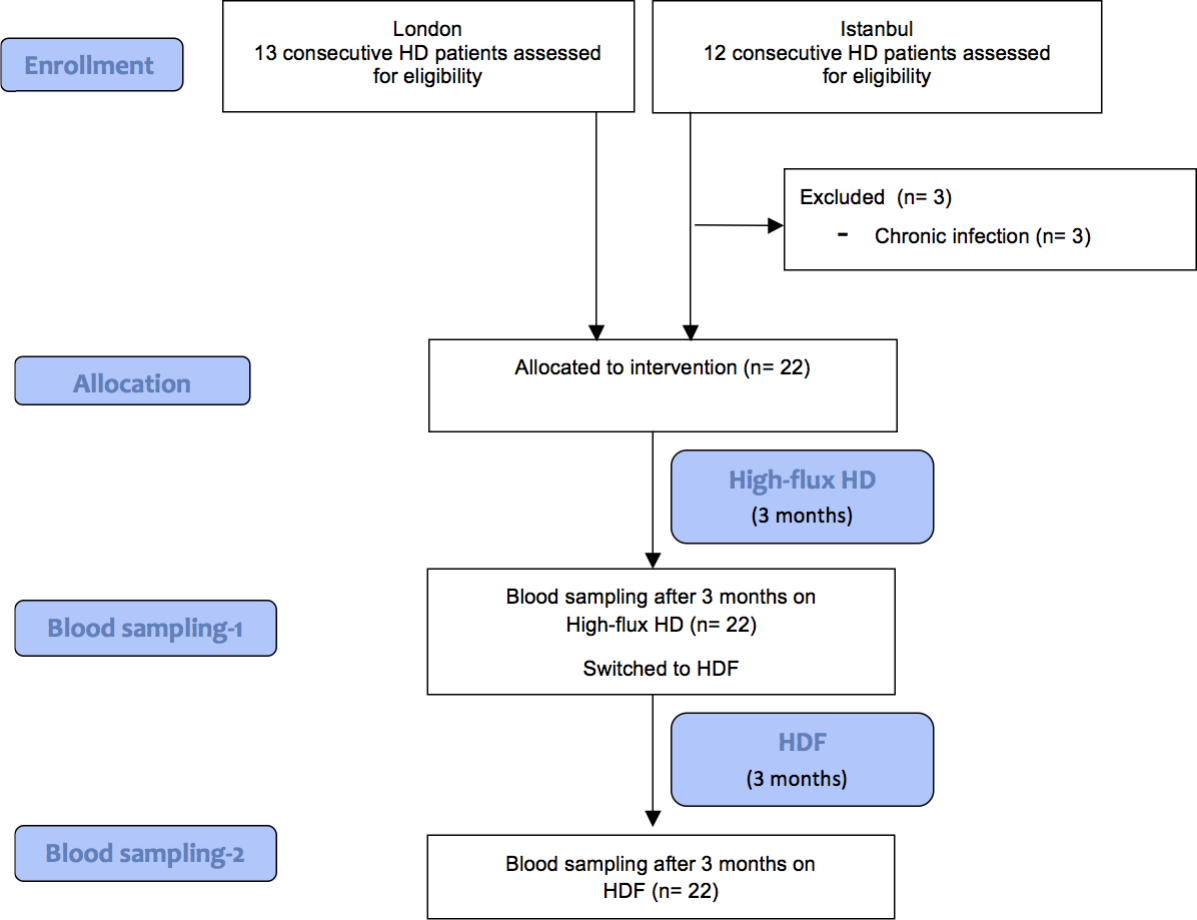
Flow chart of the study. 22 eligible chronic HD patients were recruited from two centers. Patients were treated with at least 3 months on high-flux HD and then switched to post-dilution online-HDF for 3 months. Dialysis prescription (time, blood flow rate, dialysate flow rate, dialyser type and size) and vascular access type were kept constant during each period.

All children underwent at least 3 months of regular thrice-weekly HD with high-flux membranes and were then switched to 3 months of post-dilution online-HDF (Fig 1). The dialysis prescription was kept constant during HD and HDF periods, including dialysis time (4 hours 3 times per week), blood flow rate (Qb), dialysate flow rate (Qd), and filter size and type. High-flux dialysers (Fresenius Cordiax) of the same size were used during HD and HDF periods for each child. The dialysis machines used were Fresenius 5008 in Istanbul and both Fresenius 5008 and Gambro AK200 UltraS in London. A minimum Qb of 150 ml/min/m^2^ was targeted, and a Qd/Qb ratio of 1.2 was maintained. We aimed for a target convective volume (CV), defined as the sum of replacement volume and ultrafiltration (mean value of the last 4 mid-week dialysis sessions), of 12–15 L/m^2^ body surface area, adjusting the infusion rate of the substitution fluid up to a maximum filtration fraction of 35%. Ultrapure dialysis fluids, defined as < 0.1 colony forming units per ml and < 0.03 endotoxin units per ml, were used during both HD and HDF. The water quality was checked monthly in the local laboratories and confirmed in a central lab at one time-point mid-way through the study.

### Laboratory measurements

Blood samples were collected after a minimum of 3 months on HD (HD group) and 3 months on HDF (HDF group) treatment. All samples were drawn before a mid-week dialysis session.

Besides routine biochemical parameters (urea, creatinine, electrolytes, calcium, phosphate, parathyroid hormone [*Immulite* 2500 Intact PTH assay; Siemens Healthcare Diagnostics], albumin, total cholesterol, low density lipoprotein cholesterol, high density lipoprotein cholesterol, triglycerides [all measured by colorimetric enzymatic method; *Technicon automatic analyzer RA-1000*; Dade Behring, Marburg, Germany], uric acid, hemoglobin and ferritin), markers of inflammation, oxidative stress and endothelial function were assessed. We performed the following analyses, always comparing paired patient samples (HD and HDF) and with all analyses performed by blinded investigators in a central laboratory in Istanbul University or Great Ormond Street Hospital, London: IL-6 (*e-Bioscience*, San Diego, USA), IL-10 (n = 17 samples measured, *e-bioscience*, San Diego, USA), nitrotyrosine (*Hycult Biotechnology*, Uden, Netherlands), TAC (*Cayman Chemical*, Ann Arbor, Michigan, USA), ß2M (*Siemens*, *Immulite Immunoassay system*), ADMA and SDMA (high performance liquid chromatography tandem mass spectrometry), ox-LDL (*Immunodiagnostics*, Bensheim, Germany), AGEs (*Cell BioLabs Inc*., San Diego, USA), hsCRP (*Vitros Chemistry Systems*, Ortho Clinical Diagnostics *Inc*). The % change between markers on HD and HDF was calculated by [(HDF-HD)/HD]*100 formula. Blood samples of control groups had been analyzed using the same assays as for the study population.

### Statistical analysis

Continuous data was shown as mean ± standard deviation (SD) or median (interquartile range). Within patient comparisons of biochemical measures on HD and HDF treatment were analyzed using Wilcoxon signed-rank test for median or paired sample t-test for means. Comparisons between the HD and HDF groups were analyzed using Mann-Whitney U test for medians and Chi-square test for categorical data. Univariate analysis was performed to explore associations between markers. Significant differences were defined as a two-tailed *p* value of <0.05. All statistical analyses were performed using SPSS package (version 15.0 for Windows; SPSS Inc, Chicago, IL, USA).

## Results

### Baseline characteristics

We performed a prospective observational study in 22 children on chronic HD, which were enrolled at 2 tertiary nephrology units (Fig 1). All children underwent at least 3 months of regular thrice-weekly HD with high-flux membranes and were then switched to 3 months of post-dilution online-HDF treatment. Baseline characteristics of the study population are shown in Table 1.

**Table 1:** Baseline characteristics of the patients.

*Baseline characteristics*	*Incident patients* *(n = 13)*	*Prevalent patients* *(n = 9)*	*p value*
Age, years	11.1 (7.5; 15.3)	15.3 (10.1; 15.8)	0.19
Gender (female), n (%)	6 (46)	7 (77)	0.20
Underlying kidney diseases			0.20
CAKUT, n(%)	10 (77)	4 (45)	
Glomerular disease, n(%)	1 (8)	2 (22)	
Cystinosis, n(%)	0 (0)	2 (22)	
Unknown, n(%)	2 (15)	1 (11)	
Estimated dry weight-SDS (for eight age)	0.76 (-0.74; 2.41)	-0.09 (-0.06; 0.88)	0.43
Height-SDS	-1.79 (-2.78; -0.29)	-3.50 (-7.33; -2.37)	0.006
BMI-SDS (for height age)	0.73 (-1.22; 1.76)	0.40 (-1.22; 1.53)	0.89
Dialysis vintage, months	-	36 (3.5–58)	
Urine volume, mL/kg/m2	259 (83; 454)	61 (0; 417)	0.35
HD- Blood flow rate, mL/min/m^2^ BSA	202 (189; 216)	165 (137; 216)	0.71
HDF-Blood flow rate, mL/min/m^2^ BSA	204(189; 216)	151 (131; 221)	0.71
HDF-Convective clearance, mL/m^2^ BSA	12.9 (12.2; 14.5)	12.6 (11.6; 14.8)	0.69

### Comparison of HD and HDF treatment

Dialysis related parameters on HD and HDF are shown in Table 2. All children maintained the same type of vascular access, had ultrapure dialysate, were on the same type and size of dialyser and had comparable blood flow rates, when on HD and HDF therapy.

**Table 2:** Dialysis related parameters of the patients.

	*On hemodialysis* *(n = 22)*	*On hemodiafiltration* *(n = 22)*	*p value*
Vascular access CVL/AVF/AVG, n(%)	8 (36)/ 12 (55)/ 2 (9)	8 (36)/ 12 (55)/ 2 (9)	1.00
Ultrafiltration, mL	750 (375; 1200)	600 (200; 1000)	0.87
BSA-adjusted ultrafiltration, mL/m^2^	702 (340; 1256)	635 (152; 1350)	0.54
Average blood flow, mL/min	205±64	203±66	0.18
Blood flow rate, mL/min/m^2^ BSA	192±64	190±39	0.09
Convective clearance, mL/m^2^ BSA	-	13.0±1.76	
Dialysis water quality (pure vs ultrapure)	Ultrapure	Ultrapure	
Type of dialyser	High-flux	High-flux	
Dialysis time	4 hours, thrice weekly	4 hours, thrice weekly	

Data are presented as mean ± standard deviation (SD), median (Interquartile range; IQR) or n (%).

SDS: Standard deviation score, BMI: Body mass index, CVL: Central venous line, AVF: Arterio-venous fistula, AVG: Arterio-venous greft, BSA: Body surface area.

Biochemical measures on HD and HDF therapy are shown in S1 Table. The Kt/V urea on HD and HDF did not differ significantly; 1.71±0.28 vs 1.82±0.43, p = 0.30. The hemoglobin and albumin levels were higher (11.3±1.44 vs 10.7±1.78 g/dl, p = 0.03 and 40.0±1.90 vs 37.7±3.50 g/L, p = 0.002 respectively) and uric acid levels lower (5.44±0.74 vs 6.27±1.22 mg/dl, p = 0.012) in the HDF compared to HD group. There was no difference in the dosage of iron or erythropoietin stimulating agents used during HD and HDF treatment. All other routine biochemical measures were comparable on HD and HDF.

### Markers of inflammation, oxidative stress and endothelial function on HD and HDF

**Fig 2:**
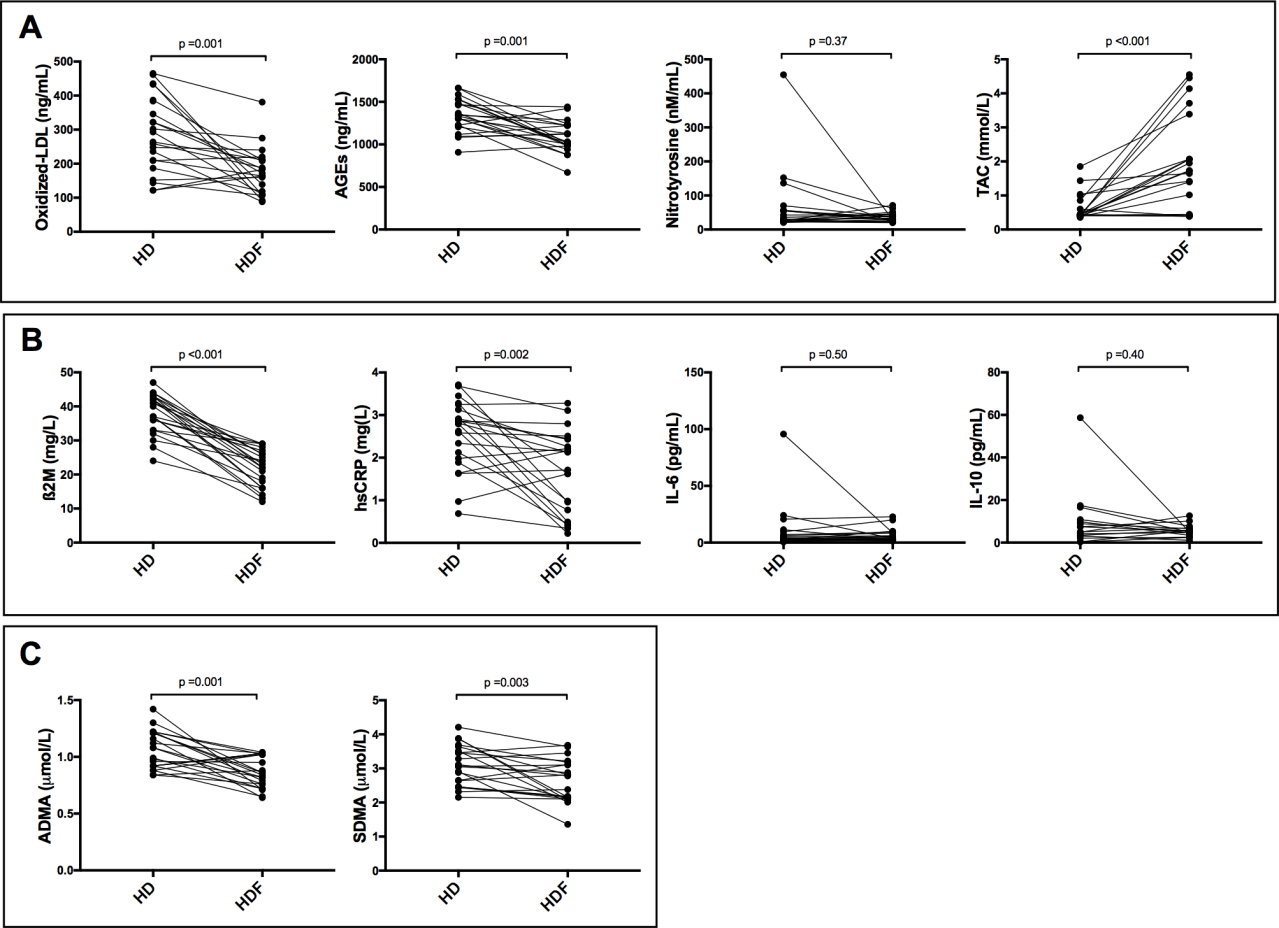
Comparison of biomarkers in children on high-flux HD and HDF. (A) Markers of oxidative stress (ox-LDL, AGEs) were significantly reduced in addition to improvement in total antioxidant capacity, however nitrosative stress (nitrotyrosine) was not changed on HDF compared to HD. (B) Markers of inflammation (ß2M, hsCRP) except IL-6 were reduced significantly while anti-inflammatory marker IL-10 was not changed on HDF compared to HD. (C) Markers of endothelial dysfunction (ADMA, SDMA) were reduced significantly on HDF compared to HD.

Switching from HD to HDF was associated with a significant reduction in β2M, hsCRP, ADMA, SDMA, AGEs, ox-LDL and a significant increase in TAC at the 3-month assessment (Fig 2; S3 Table). The reduction in hsCRP positively correlated with the reduction in ADMA and SDMA (r = 0.52, p = 0.01; r = 0.54, p = 0.009 respectively). Nitrotyrosine, IL-6 and IL-10 did not change significantly after switching to HDF (p = 0.37, p = 0.50, p = 0.40, respectively).

After 3 months of HDF, all the biomarkers were comparable between incident and prevalent patients except IL-10 levels which were significantly lower in the prevalent group (p = 0.01; S2 Table), but did not correlate with dialysis vintage.

### Factors influencing markers of inflammation, oxidative stress and endothelial function

#### I—Dialysis related factors

**Fig 3:**
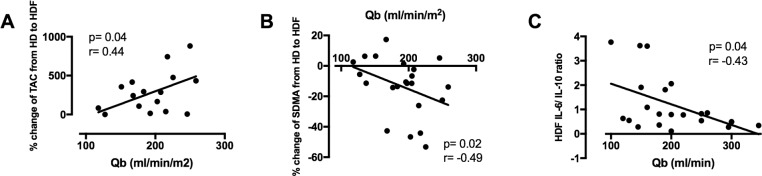
Percent increase in TAC and percent decrease in SDMA from HD to HDF was more pronounced in patients with higher blood flow rate (A,B). Higher blood flow rate was also associated with lower IL-6 to IL-10 ratio on HDF (C). Percent change = [(HDF-HD)/HD]*100.

The type of vascular access did not influence any of the markers studied. On univariate analysis a higher blood flow rate (standardized for body surface area) was positively associated with the percentage change in TAC and negatively associated with the percentage change in SDMA levels (r = 0.44, p = 0.04; r = -0.49, p = 0.02, respectively; Fig 3A and 3B) for intra-individual changes from HD to HDF. Patients with a higher blood flow had lower IL-6/IL-10 ratio on HDF (r = -0.43, p = 0.04; Fig 3C).

A target convective volume (CV) of 12–15 L/m^2^ body surface area, which is comparable to 20–25 L per session in an adult, was aimed for in all HDF patients. The median time-integrated CV over the 3-month period that was achieved was 12.8 (12.1; 14.7) L/m^2^. Target CV was achieved in 17 (77%) children, and ranged from 9.3 and 11.9 L/m^2^ in the remaining 5 children; this was unrelated to access type, but largely due to lower blood flows. Children with a convective volume above 12.8L/m^2^ (the median level achieved) had significantly lower ox-LDL (114 [103; 185] vs 208 [164; 240] ng/mL, p = 0.02) compared to those who achieved a lower CV.

#### II—Incident vs prevalent HD patients

There were no significant difference between incident and prevalent patients in age, gender, underlying disease, residual urine volume, or dialysis related measures such as vascular access type, blood flow rate or convective volume achieved (Table 1). Prevalent patients had a significantly lower height SDS compared to incident patients. There was no difference in any of the biomarkers of inflammation, oxidative stress or endothelial function between incident and prevalent HD patients (S2 Table). IL-10 levels were higher in incident (n = 13) compared to prevalent (n = 9) patients on HD (8.9 [4.9; 17.4] vs 4.1 [1.2; 8.6] pg/mL respectively, p = 0.06), and this effect persisted when these patients were moved to HDF (6.5 [5.2; 11.9] vs 4.4 [2.5; 6.3] pg/mL respectively, p = 0.01). There were no significant differences between incident and prevalent patients in any other biomarkers, suggesting that even a short dialysis vintage of 3 months on HD leads to increased inflammation and oxidative stress and a deterioration in endothelial markers.

#### III—Urine volume

**Fig 4:**
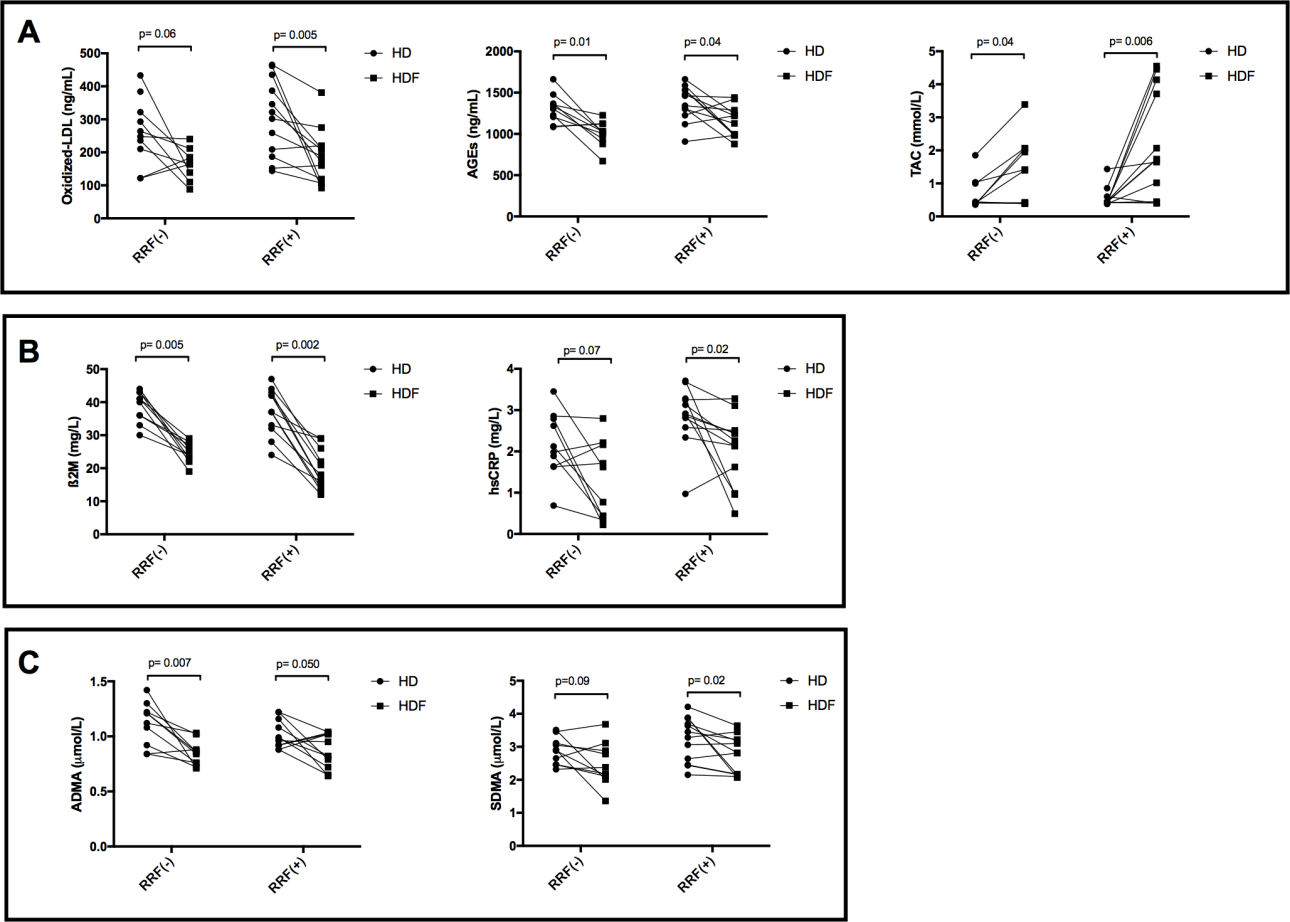
Comparison of biomarkers in children with and without residual renal function (RRF) on high-flux HD and HDF. Markers of oxidative stress (A), inflammation (B) and endothelial dysfunction (C) were significantly decreased in addition to a significant increase in anti-oxidant capacity on HDF compared to HD. This improvement was observed not only in patients without RRF but also in patients with RRF.

We examined the effect of residual renal function (RRF), defined for the purpose of this study as a urine volume ≥ 200 ml/day, on biomarkers in the HD and HDF groups (Table 3). There was no statistically significant difference between children with (n = 12) and without (n = 10) RRF concerning body surface area (p = 0.71) and achieved convective volumes (12.8±1.8 L/m^2^ vs 13.2±1.8 L/m^2^, p = 0.60). After switching to HDF, ß2M levels decreased by 34 (48.0; 24.3)% in children without RRF, and by 51 (62.0; 35.2)% in children with RRF (p = 0.06). Even in children with RRF, HDF achieved a significant reduction in hsCRP, AGEs, ADMA, SDMA (p<0.05 for all), ß2M and ox-LDL (p<0.01) levels, and improvement in TAC (p<0.01) compared to HD (Table 3; Fig 4).

**Table 3:** Comparison of the inflammatory, oxidative stress and endothelial markers between HD and HDF in patients with and without residual renal function.

	*Urine volume <200 ml/day (n = 10)*				*Urine volume ≥200ml/day (n = 12)*			
	*HD*	*HDF*	*p*^*1*^	% change from HD to HDF*	*HD*	*HDF*	*p*^*2*^	% change from HD to HDF*
Ox-LDL, ng/mL	256(188;337)	164(124;198)	0.06	-21.9(-62.5;15.6)	305(192;423)	186(110;217)	0.005	-31.4(-49.0;-11.2)
AGEs, ng/mL	1318(1177;1395)	1020(926;1121)	0.01	-25.2(-36.5;-6.34)	1371(1244;1531)	1149(985;1276)	0.04	-15.3(-34.4;5.73)
TAC, mmol/L	0.41(0.39;1.01)	1.40(0.40;2.06)	0.04	82.9(-1.1;329)	0.55(0.42;0.56)	2.19(0.58;4.03)	0.006	289(7.61;665)
ß2M, mg/L	40.5(35.2;43)	24.5(22.7;27.2)	0.005	-34.1(-48.0;-24.3)	37.1(32.2;42)	19.3(14.5;25)	0.002	-51.5(-62.0;-35.2)
hsCRP, mg/L	2.05(1.63;2.80)	1.19(0.38;2.17)	0.07	-51.8(-78.9;6.5)	2.86(2.63;3.27)	2.03(1.14;2.49)	0.02	-15.9(-56.6;-4.17)
IL-10, pg/mL	4.18(2.08;8.93)	5.60(2.54;11.7)	0.90	11.0(-64.0;156)	7.42(4.24;17.0)	6.50(4.90;7.48)	0.26	-25.0(-64.0;54.0)
ADMA, μmol/L	1.16(0.9;1.24)	0.85(0.75;0.91)	0.007	-25.3(-31.3;-9.15)	1.01(0.92;1.14)	0.87(0.73;1.02)	0.05	-15.5(-26.9;11.6)
SDMA, μmol/L	2.89(2.46;3.19)	2.28(2.08;2.93)	0.09	-10.9(-30.2;3.52)	3.23(2.49;3.82)	2.74(2.16;3.21)	0.02	-11.4(-20.3;0.39)

Data shown as median (IQR); Mann Whitney U test

* % change from HD to HDF was calculated with [((HDF-HD)/HD)*100] formula. No significant difference was found between two groups for % change from HD to HDF

HD: Hemodialysis, HDF: Hemodiafiltration, Ox-LDL: Oxidized Low density lipoprotein, AGEs: Advanced glycation end-products, TAC: Total antioxidant capacity, ß2M: Beta 2 microglobulin, hsCRP: High sensitive C-reactive protein, IL: Interleukine, ADMA: Asymmetric dimethyl arginine, SDMA: symmetric dimethylarginine

## Discussion

We have shown that HDF is associated with reduced biomarkers of inflammation, oxidative stress and endothelial dysfunction and improved antioxidant capacity compared to conventional HD in children. Incident patients on HD for only 3 months had a comparable biomarker profile to those on chronic HD for several years, suggesting that HD causes early onset changes. However, within only 3 months of switching to HDF, keeping all other dialysis related parameters constant, significant improvement was seen in several markers of inflammation, oxidative stress and endothelial dysfunction, implying that convective clearance plays a crucial role in improving the biomarker profile. The blood flow rate and convective volume were important modifiable factors that influenced this improvement. Children with residual renal function also had a significant but attenuated improvement in their biomarker profile.

The proposed mechanisms of reduced inflammation, oxidative stress and endothelial dysfunction on HDF compared to HD are biocompability and enhanced clearances across a wide molecular weight range. By using an ‘ultra-pure’ dialysis fluid and biocompatible membranes, the generation of inflammatory mediators is theoretically reduced. In the present study we used ultrapure dialysate and the same type of high-flux membrane for all children throughout the HD and HDF periods, aiming to eliminate the possible effect of biocompability on the improved biomarker profile. However, studies in adult dialysis patients that have used ultrapure dialysate and the same membranes on both high-flux HD and HDF treatment arms, have also shown reduced generation of inflammatory mediators such as lower circulating pro-inflammatory monocytes (CD14+CD16+) and TNFα mRNA on HDF [14,20,21]. The CONTRAST study that randomized patients to HDF vs low-flux HD has shown that over the 3-year trial period CRP and interleukin-6 concentrations increased in patients treated with HD, and remained stable in patients treated with HDF, suggesting that long-term HDF with ultrapure dialysate seems to reduce inflammatory activity over time [22].

**Fig 5:**
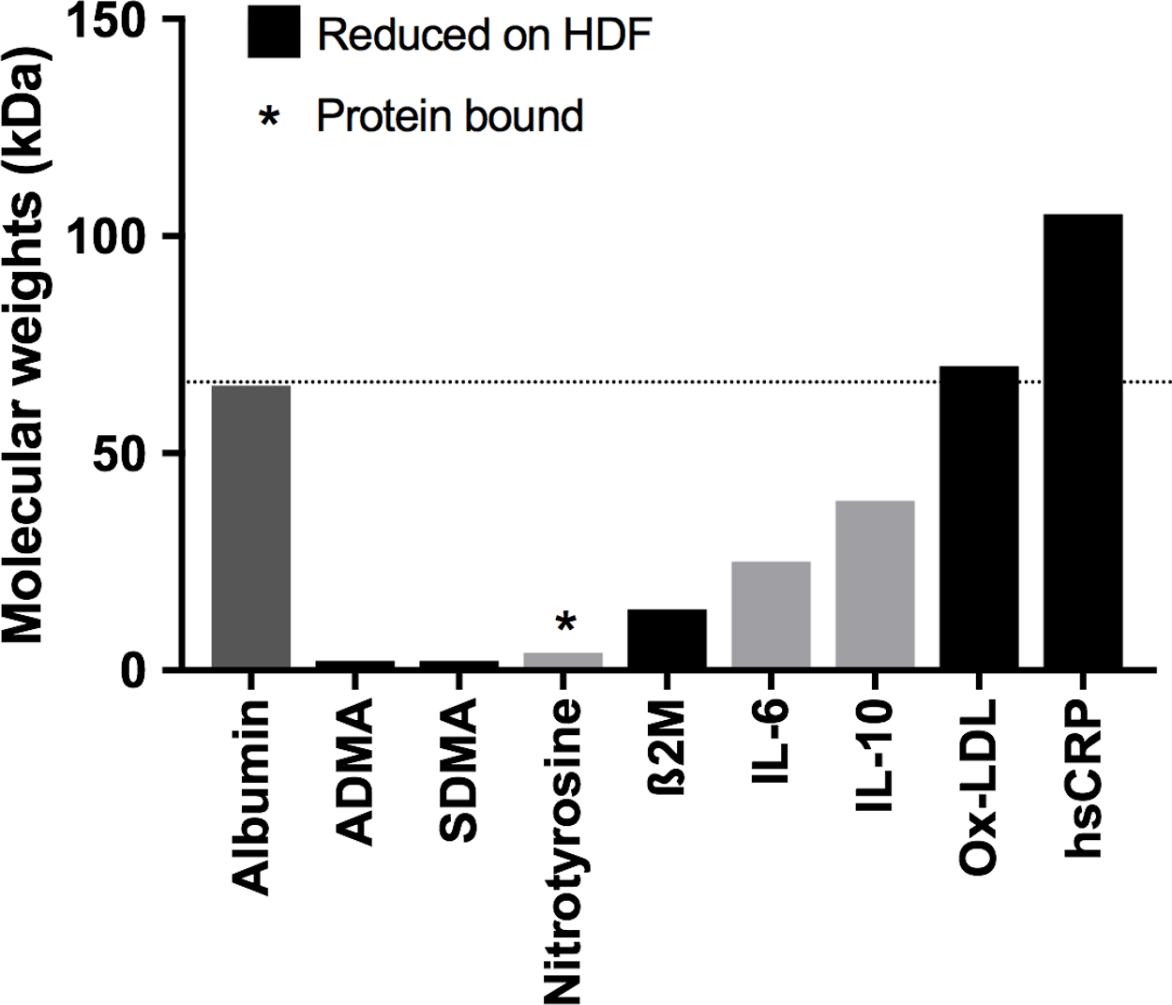
Demonstrating the molecular weights of the studied markers and comparison with albumin. AGEs are not shown in the figure because they are composed of several molecules, which are mostly middle molecules.

A second important advantage of HDF is the enhanced removal of middle molecules by convection [9,23]. Substances that have molecular weights lower than albumin can be removed by convection, but this is strongly influenced by the mass transfer coefficient of the dialysis membrane [24] and the convective volume applied [25]. Large randomised trials in adults have shown improved cardiovascular and survival outcomes only when high convective volumes in the range of 17–20 L per session were used in post-dilution HDF [12,13,26]. Extrapolating from these data, we aimed for convective volumes of 12–15 L/m^2^ and we were able to achieve this in 77% of children. Interestingly, although ox-LDL and hsCRP have a higher molecular weight than albumin (Fig 5), their levels were significantly lower on HDF compared to HD, with lower ox-LDL levels associated with higher convective volumes. Indeed, other studies have also shown that high volume HDF using high fluid substitution rates allows a greater clearance of large uremic toxins [27]. We did not find any correlation between convective volume and other biomarkers, probably because a high convective volume was achieved in most children. Notably, none of the children on HDF had low albumin levels, dismissing the fear that HDF through high-flux filters can cause albumin loss. Higher blood flow rates were associated with reduced inflammation (IL-6/IL-10 ratio), a greater decrease in SDMA and increase in TAC in children on HDF compared to HD, keeping in mind that the same access type and blood flow rate was maintained in each child on HD and HDF. This suggests that for a given blood flow HDF achieves superior clearances compared to HD.

Residual renal function plays an important role in the removal of uremic toxins. ß2M with a molecular weight of 11.8 kD is widely studied as a marker of middle molecular weight uremic toxins, and causally associated with dialysis related amyloidosis [28]. ß2M is almost exclusively eliminated by the kidneys and dialysis patients with even minimal RRF can have some clearance of ß2M [29], conferring a survival advantage [30]. In our cohort, ß2M and several other middle molecules that are biomarkers of oxidative stress, inflammation and endothelial dysfunction were significantly lower on HDF compared to HD even in patients with RRF. The CONTRAST study showed that serum ß2M levels decreased after 6 months of treatment with HDF but slightly increased in patients on low-flux HD [31]. Interestingly, both our work and the CONTRAST study showed that the amount of convective volume was not related to the decrease in ß2M levels [31] whereas the degree of RRF was shown to correlate in the CONTRAST study. This may be explained by the multi-compartmental distribution of ß2M: the removal rate by dialysis is almost similar to the transfer rate from the tissues into the vascular compartment [32], implying that other dialysis strategies such as increased treatment time or more frequent dialysis may be required. We also found that other middle molecules like AGEs and even some large molecular weight substances like ox-LDL, hsCRP had lower levels on HDF compared to HD. Ox-LDL and hsCRP have molecular weights that are larger than that of albumin, so their clearance is very unlikely, but removal of smaller molecules that trigger ox-LDL and hsCRP formation may have led to an improved antioxidant–oxidant balance and reduced levels of these molecules on HDF. Taken together, our data suggests that HDF achieves superior removal of middle molecules compared to HD even in those with RRF and should be the preferred dialysis modality irrespective of RRF.

Interleukin-6 and IL-10, that are pro-inflammatory and anti-inflammatory cytokines respectively, are middle molecules and thus expected to be removed in HDF. However, different studies have reported conflicting results, with some reporting reduced levels IL-6 [19,21,33], and increased IL-10 [33], whereas others demonstrate no significant decrease on HDF compared to HD [34,35]. We were unable to show a difference in IL-6 and IL-10 levels between HDF and HD groups, despite using high convective volumes, and postulate that high biocompatibility achieved during HD therapy may have reduced the generation of IL-6.

There are no published data about the effect of HDF on oxidative stress in children. We found that some markers of oxidative stress including ox-LDL and AGEs were reduced and TAC was increased in HDF compared to HD group. Studies in adults on HDF have demonstrated reduced markers of oxidative stress such as decreased serum ox-LDL [36], AGEs levels [35,37], advanced oxidation protein products [35] and improvement in antioxidant status as demonstrated by increased TAC [34]. TAC measures the total effect of anti-oxidants, which include low, middle and large molecules. Even though low and middle molecular weight antioxidants are removed, increased TAC may indicate increased production or reduced consumption of antioxidant molecules. Reactive nitrogen species, assessed in our study with nitrotyrosine, are a component of oxidative stress, and are largely protein-bound molecules. Increased nitrosilation of plasma proteins has been shown in HD patients [38], and are not cleared by any dialysis modality.

ADMA and SDMA are endogenous inhibitors of nitric-oxide synthase leading to increased systemic vascular resistance. Elevated ADMA and SDMA are related with increased all-cause mortality and cardiovascular disease [39]. Both markers accumulate in chronic kidney disease by increased synthesis, reduced catabolism and reduced renal clearance [40]. They are small molecules (202 Da) but poorly dialyzed with a substantial rebound occurring at the end of dialysis, which is explained by extended volume of distribution [41,42]. In our study ADMA and SDMA were significantly reduced after 3 months on HDF treatment. A reduction in hsCRP was positively correlated with reduction in ADMA and SDMA in HDF, suggesting that reduced generation, increased catabolism as well as increased clearance may account for a reduction in these markers. Also this reduction in ADMA and SDMA may be related with reduced oxidative stress and inflammation. Ox-LDL and AGEs are markers of oxidative stress but also play a direct role in the pathogenesis of endothelial dysfunction, and were also significantly reduced in our study. An improvement in the endothelial marker profile on HDF treatment has shown by other studies in adults; reduced ox-LDL [36], better NO availability [14], improvement in endothelial damage-repair balance [20], lower pulse wave velocity [43], increase in carotid artery distensibility and brachial flow mediated dilatation [14].

It is suggested that ESKD patients who are treated exclusively with HDF and never exposed to HD have improved survival [44]. In our cohort, after 3 months of HD, levels of the studied markers did not differ between incident and prevalent HD patients, indicating even 3 months on HD is associated with an unfavorable biomarker profile.

The strength of our study is evaluating the effects of HDF on three closely related systems: inflammation, oxidative stress and endothelial dysfunction using a wide range of biomarkers in children. None of the children in our study had diabetes or pre-existing cardiovascular or peripheral vascular disease, none were smokers and none had autoimmune disease or vasculitides. Thus, confounders for inflammatory and endothelial disease were eliminated and we were able to study the effects of different dialysis modalities in a ‘clean’ model. Although we noted an adverse biomarker profile within 3 months of chronic HD, we are not able to determine the impact of CKD and whether HD treatment caused a further deterioration or even an improvement of these biomarkers. Due to the short study duration, we cannot correlate changes in the biomarker profiles with vascular function such as carotid distensibility, pulse wave velocity or flow mediated dilatation in this short time period. Since the duration of the HDF period was only 3 months, we do not know if the improvement in biomarker profile would be sustained over longer periods, or if the markers that were unchanged in 3 months may improve with time. To explore these issues we have designed a longitudinal follow-up study across Europe that measures changes in vascular structure and function in children on HD and HDF over 1 year [45]. Although incident and prevalent patients were from different centers, we were not able to find any significant differences between the groups in any demographic, dialysis-related parameters or medication, but prevalent children on HD were significantly shorter. We were unable to perform a full cross-over trial design as many children felt substantially better on HDF and refused to return to HD. We do not have data on biomarker levels in healthy controls, but since the study design looks at the intra-individual change in biomarkers between dialysis modalities this was not essential. Finally we did not calculate the creatinine clearance, but instead used daily urine volume as a surrogate to define RRF.

## Conclusions

We have shown that a significant improvement in inflammation, antioxidant capacity and endothelial risk profile is achieved even within a short time (3 months) on HDF compared to high-flux HD, and that this effect is seen even in children with residual renal function. Longitudinal studies are required to determine if this improvement in biomarker profile translates to improved vascular function and survival.

## Supporting information

S1 checklist CONSORT checklist. (PDF)

S1 protocol The study protocol. (PDF)

S1 Table Comparison of the biochemistry data of the patients on HD and HDF. (PDF)

S2 Table Comparison of the inflammatory, oxidative stress and endothelial markers between HD and HDF in incident and prevalent patients. (PDF)

S3 Table Comparison of the inflammatory, oxidative stress and endothelial markers between HD and HDF. (PDF)
