# Supplementary material for: Hemodiafiltration is associated with reduced inflammation, oxidative stress and improved endothelial risk profile compared to high-flux hemodialysis in children
Source: PLoS One. 2018 Jun 18;13(6):e0198320. doi: 10.1371/journal.pone.0198320 (PMC6005477; doi:10.1371/journal.pone.0198320)
Supplement: S1 protocol — (PDF) [file pone.0198320.s002.pdf]

Effects of HD and HDF on inflammation, endothelial dysfunction and oxidative stress in children - SWITCH STUDY

Principal Investigator	Dr. Rukshanna Shroff
Co-investigators/ Site PI	Dr. Nur Canpolat, Istanbul
Document type	Study Protocol
Version number	Version 1.0
Release date	20 May, 2015
Principal Contact	Dr. Rukshanna Shroff<br>Great Ormond Street Hospital<br>London, England<br>Rukshana.Shroff@gosh.nhs.uk

Table of Content

İntroduction

- Study Abstract..........Page 3
- Inclusion/Exclusion Criteria..........Page 3

Materials/Methods..........Page 3

Identifying potential study Subjects..........Page 4

Ethical Considerations..........Page 5

Informed Consent Process

- Risk and Benefits..........Page 6

Health Related Questionnaires

- Quality of Life (QoL) Questionnaires..........Page 7
- Case Report Forms and Source Documents..........Page 7
- Records Retension..........Page 8

Organization and Participating Centers..........Page 8

Data Monitoring Commitee..........Page 8

Data Storage

- Confidentialy and Security..........Page 8

Samples

- Blood Sampling..........Page 9
- Specimen Handling and Storage..........Page 10

Time Line..........Page 11

List of Markers

- Oxidative Stress..........Page 11
- Endothelial Markers..........Page 12

References..........Page13

SWITCH STUDY PROTOCOL

INTRODUCTİON:

Study Abstract:

A prospective multicentre study.

Hemodiafiltration can remove middle molecular weight uremic toxins like cytokines with convection method while hemodialysis mostly removes low molecular weight toxins by diffusion method. Hemodiafiltration is thougt to cause less inflammation and oxidative stress with ultrapure dialysate and biocompatible membranes. Currently, no studies in pediatric population involve investigating the difference in oxidative stress and inflammation between these dialysis modalities.

The aim of this study is to investigate the alteration of markers of oxidative stress/inflammation between different dialysis modalities ie; hemodialysis versus hemodiafiltration.

Inclusion criteria:

- Incident or prevalent HD patients
- Under 18 years old

Exclusion criteria:

- Any systemic disease with active inflammation ie; active period of vasculitis
- İmmune deficiency
- Patients using İmmunosuppresive drugs
- Any patients with chronic inflammation
- Smokers
- Patients with diabetes
- Patients with pre-existing cardiovascular disease

MATERIALS AND METHOD:

- Type of vascular access (fistula vs central venous catheter), surface area of the dialyzer, blood flow rate, time and frequency of the dialysis sessions. .
- Standardization of the prescription between centers;

	HD	HDF
Dialyzer	highflux membranes; cordiax	highflux membranes; cordiax
Dialyzer surface area	Body surface area ±%20	Body surface area ±%20
Qb	≅ 150 ml/dk/m2	≅ 150 ml/dk/m2

	Qb/Qd: 1.5-3	UF rate: postdilution-1/3Qb

IDENTIFYING POTENTIAL STUDY SUBJECTS:

All children on HDF type of dialysis enrolled in HDF-3H study are eligible for participating in the SWITCH study. Potential participants will be approached in the haemodialysis unit or in pre-dialysis CKD clinics by the clinicians who look after them at each site.

All samples will be anonymised using a study code that is known only to the local Principal Investigator and their designated researchers. No persons in the central laboratory will be able to link any biochemical data with individual patients. Samples will be centrifuged and the serum stored at -80°C locked freezers in the local hospitals. Cellular material from blood samples will not be required. Of note, no genetic tests will be performed in the course of this study. Only the serum samples (and not any cellular material) will be batched and transferred on dry ice using designated approved couriers to the Chemical Pathology Unit, Great Ormond Street Hospital, London. At Great Ormond Street Hospital, samples will be stored in locked freezers at -80°C until analysed. At the end of the study period samples will be stored for a further 15 years in -80 freezers at the ICH. The samples will be stored pending ethical approval for another study. Dr Shroff will hold custodial rights to all the samples.

All investigations will be performed in a central laboratory at Great Ormond Street Hospital.

These questionnaires will be sent as one 'set' to the local teams to distribute to each family. Completed questionnaires will be sent to the Research Psychotherapist, Dr Claire Dempster, who will be responsible for ensuring that questionnaires are screened within two weeks of receipt and any concerning information is fed back to the relevant study site as appropriate. Where the questionnaires indicate high levels of clinical concern about either mental health issues or quality of life, a report will be sent to the Chief Investigator, Dr Shroff, and to the young person's Consultant for further action in order to ensure that any risk or safety planning can take place as required. It will be the responsibility of the local team to manage the psychosocial concerns as they see appropriate and with the help of their local psychosocial teams. All questionnaires will be fully anonymised and only the local PI will be aware of the research code and will be able to identify the patient.

The Research Assistant will help with the distribution, collection of completed questionnaires, ensuring that questionnaire data sets are checked, anonymised and the database is maintained. Funding has been allocated for translation of questionnaires into the child or young person's language as required. Similarly this funding will be used to translate any questionnaires for interpretation as necessary.

Samples will be transferred from Paediatric Dialysis centres in the UK and the study sites in the EU as listed below:

The UK sites are:

1. Evelina Children's Hospital, London

2. Queen's Medical Centre, Nottingham

3. Birmingham Children's Hospital, Birmingham

4. University Hospital of Wales, Cardiff

5. Bristol Children's Hospital, Bristol

6. Alder Hey Hospital, Liverpool

7. St. James's University Hospital, Leeds

8. Manchester Royal Infirmary, Manchester

9. Royal Hospital for Sick Children(Yorkhill),Glasgow

10. Southampton General Hospital, Southampton

11. Newcastle Upon Tyne Hospital, Newcastle

European sites that are part of the study are in Germany, France, Poland, Austria, Italy, Switzerland, Czech Republic, Finland, Sweden, Lithuania and Latvia.

ETHICAL CONSIDERATIONS

The study will be conducted in accordance with the International Conference on Harmonization (ICH) Guideline for Good Clinical Practice (GCP) all applicable subject privacy requirements and the guiding principles of the Declaration of Helsinki. Conduct of the study includes, but is not limited to, the following:

• Institutional Review Board (IRB)/Independent Ethics Committee (IEC) review and favorable opinion/approval of study protocol and any subsequent amendments.

• Subject/subject's parent(s) informed consent and subject informed assent, as appropriate.

• Investigator reporting requirements as stated in the protocol.

Freely given and written informed consent must be obtained from each subject and/or each subject's parent(s) and subject informed assent, as appropriate, prior to participation in the study. Those subjects who can only be enrolled in the study with the consent of the subject's LAR (legally acceptable representative) (e.g. minors), should be informed about the study to the extent compatible with the subject's understanding and, if capable, the subject should sign and personally date a written informed assent. It is required that the assent be signed by each

subject, if capable, in addition to the informed consent that is to be signed by his/her legal representative. It should be assessed whether an assent is required depending of the age of the study population and the local requirements.

The study protocol, questionnaires, and consents, will be reviewed and approved by the appropriate REB.

INFORMED CONSENT PROCESS:

Informed consent will be sought from participants themselves or their parent/legal guardian on behalf of their child. Children who do not have the capacity to consent for themselves will sign an assent form in addition to their signed parental consent.

In order to ensure participant's rights are protected, those who sign an assent form will be flagged and their capacity to consent will be assess annually at regular clinic visits. When a participant is deemed to have the capacity to consent for research study, a study team member will approach the patient to obtain their consent on their continued participation in the long-term storage of their biosamples for the study. The participant will be given a detailed explanation of the reason of re-approaching them for consent and all questions will be answered to their satisfaction. Participants will be given a choice if they would like to continue participating in the study by allowing long-term storage of their collected biosamples, or if they would like to withdraw their samples from the study.

Participants will be asked to sign the consent form to indicate their preferences of continued participation and will be given a copy of their signed consent form for their records. Participants who would like to withdraw their samples will be noted, and samples will be withdrawn from the biobank.

Study will be kept open for up to 15 years post study closure (data collection ended) to ensure all participants who acquired capacity to consent for themselves are approached.

Risks and Benefits:

Potential Risks: Blood samples are collected as part of standard of care pre and post-transplant. We will be collecting the data obtained from the tests performed.

Potential Benefits: There may or may not be direct benefit to participants. We anticipate that the information learned from this study will benefit other children on HDF type of dialysis.

HEALTH-RELATED QUALITY OF LIFE QUESTIONNAIRE (BASELINE AND 6-MONTHLY INTERVALS):

Three information and screening questionnaires will be given to children and their parents: Pi-ED will screen for emotional distress (depression and anxiety), PedsQL for health impacts on QoL and SDQ for emotional well being and pro-social relationships. In addition, we will acquire information on post-dialysis recovery time, school and college attendance and sleep pattern.

1. PedsQl (Paediatric Quality of Life)

2. SDQ (Strengths, Difficulties Questionnaire)

3. Pi-Ed (Paediatric Index of Emotional Distress)

Quality of life (QoL) questionnaires:

Three validated questionnaires:

PedsQL (Pediatric Quality of Life)

SDQ (Strengths-Difficulties Questionnaire

Pi-ED (Paediatric Index of Emotional Distress) will be used.

Case Report Forms and Source Documents:

All data will initially be collected on paper CRF's at each site. The site investigator must retain a copy of the CRF and changes in the study files. Subject data necessary for analysis and reporting will then be entered/ transmitted into a validated database or data system.All data will be entered in the HDF-3H Registry (http://pedpd.org/index.php) only – no separate data entry required. Clinical data management will be performed in accordance with applicable International Child Health standards.

Data required (all part of the HDF-3H study)

(note, all baseline data will be available from HDF-3H)

- On the day of assesment rule/out any infection, inflammation with symptoms, physical examination (including and examination of vascular access about hyperemia, purulent discharge) and laboratory (leukocytosis, CRP, urine analysis - if patient is not anuric).
- Antropometic measurements (dry weight, height)
- Type of vascular Access, dialysis modality, machine, Qb,Qd, replacement volume, dialyzer, dialysate will be recorded

- Kt/v, routine blood tests (hemogram, urea, creatinin, uric acid, electrolytes, HCO3, lipids (optional), ferritine, PTH) , drugs will be recorded from patients' files

Records Retention:

Hard copies of participant data records (source documents) will be retained on site and at the coordinating center for 7 years after the last study follow up is completed. Electronic data in the centralized research database will be retained for up to 20 years after the last study follow up is completed and analyses related to the principal research or ancillary studies has been completed. After this point, written copies of the research records will be destroyed. Information retained (electronic and hard copy) that links the electronic database with individual participants will also be destroyed or deleted.

ORGANIZATION AND PARTICIPATING CENTERS:

The study will be coordinated centrally. The coordinating center will include the multicenter research coordinator who will manage communication with sites. The site research nurse at the coordinating center will also provide oversight over standard operating procedures, and contribute to training site research associates/nurses. The core site coordinator and multicenter research coordinator will be accountable to the principal investigator.

Each center will have one designated site investigator who will be responsible for the conduct of the study at their site, and will employ a research nurse/associate to complete the site study visits. The site research associate will be responsible for completing study visit, collecting data at the visit and complete data collection for results that come after the visit, for sample collection, processing and storage.

Data Monitoring Committee:

A Data Monitoring Committee will oversee the study and interim analysis.

DATA STORAGE:

Confidentiality and Security

The study will be conducted in accordance with the ethical principles of the Declaration of Helsinki and will be consistent with Good Clinical Practices (GCP) and applicable regulatory requirements under PHIA . Each site will take appropriate measures to ensure the confidentiality of the data and the well-being of their participants. Information gathered in this research study may be published or presented in public forums; however participant names and other identifying information will not be used or revealed. Organizations such as the local Research Ethics board may inspect and/or copy research records for quality assurance.

Individuals who will have access to records containing personal health information such as hospital medical records will be restricted to the investigators of the study and study personnel associated with the trial.

Participants in the study are assigned a unique study identification number that is linked at the site only. This identification number is the only patient identifier stored with the research data. The participant is not tracked through the study by name, social security number, medical record number, or other distinct personal identifiers. A log of the participant names and participant ID numbers as well a sample code and participant ID will be maintained in a locked area at each clinical site. The participant and sample log will remain at the site of origin. Any communication between the coordinating centers and individual sites will occur via the participant or sample ID number. Any forms or documents sent to the clinical coordinating centers, IRB or Regulatory Authorities will have all personal identifying information removed, and replaced with the participant ID number. All research reports, articles, and presentations will report only aggregate findings.

The hardware will reside within a restricted access area managed by the Computer Operations group and site administrators will have remote access to the servers. Access to the content or to any components of the database will require login authentication (i.e., username and password). The privacy of the participant content is ensured and managed by the database manager. Connection to the site will be over HTTPS (secured socket layer) and therefore all data entered will be encrypted and trusted.

The electronic database will not contain personal health or otherwise identifying information. The database will be permanently de-linked from identifying information after site linking data has been deleted. Thereafter, stored data may be used for supplementary analyses as anonymized data. At this point, it will no longer be possible to collect or include additional patient-specific information.

SAMPLES:

Blood sampling:

- At baseline and 3 months (= end of study)
- 18 ml blood; to be collected from the patient's vascular access just before they start dialysis. Please collect the sample on a mid-week session of dialysis.
- Please collect using the 3H study packs provided.
- Centrifuge and save serum in 10 bottles and store at -80 degrees
- Courier to the central laboratory in Heidelberg (exactly the same as for 3H samples)

Specimen Handling & Storage

As part of the main study, blood samples are stored for testing. We may not use the entire sample collected in this research study. We will keep the sample, for possible use in other research. Future research studies may be performed by the study collaborators, the research team doing this study and our research staff, involved in kidney disease research. The Investigator at the coordinating centre will take responsibility for sending samples to researchers who work with this team.

Participants can refuse Consent for use of blood samples in future research, but still consent to the proposed study. If participants refuse to have their blood stored for future research their blood samples will be destroyed at the end of this study.

The stored samples will be labeled by study number only with no identifying information. After the study is complete the samples that are stored will be linked to study data using a unique identifier but not to participant's identity. Only the Investigator at participating sites and his/her research staff will have access to the participants' identifying information which will be kept in a locked research space at this centre. Any identifying information on paper or stored on a computer (password protected) will be in a locked office and will be accessed only by research staff at this site. We will not be able to give out any information that identifies the participant, to the scientists who receive the samples.

At any time participants may ask to stop allowing the storage of their samples. If a decision is made to stop allowing the use of stored samples, they will be destroyed. Information and results already collected up to that time will be kept in the study information files.

Please see the "Goverence of Biospecimen" attached.

TİME-LİNE:

Baseline – At the time of study entry when the first blood samples for the study are collected. This is the same as the bloods for the 3H study.

End of study (3 months of the new dialysis modality) - Assesment will be done at the end of 3 months of HDF

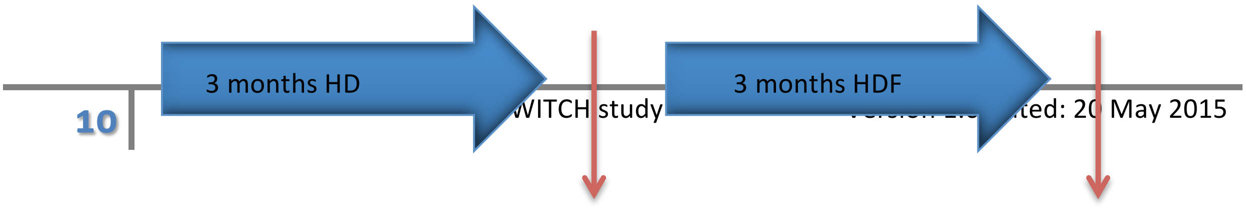

Time "0"

Blood sampling

IPHN data entry registry

"Final"

Blood sampling

IPHN data entry registry

LIST OF MARKERS

OXIIDATIVE STRESS:

LIPIDS:

- Oxidized LDL
- Lipid hidroperoxide

PROTEINS:

- Nitrotyrosine

ANTIOXIDANTS:

- Gluthatione peroxidase
- SOD

INFLAMMATORY MARKERS:

- LP-PLA2
- Pentraxin 3
- IL 6
- IL 10
- TNF alpha
- hsCRP

- ADMA
- AGEs
- B2 microglobulin
- PTH

ENDOTHELIAL MARKERS

1. Nitric oxide
2. ADMA and SDMA

3. oxLDL
4. Endothelial ROS production
5. Microparticles (endothelial, platelet, leukocyte)
6. iCAM and VCAM
7. HDL function (including NO production) in a sub-group

Reference List

(1) Blankestijn PJ, Ledebo I, Canaud B. Hemodiafiltration: clinical evidence and remaining questions. Kidney Int. 2010; 77(7):581-587.

(2) Ledebo I, Blankestijn PJ. Haemodiafiltration-optimal efficiency and safety. NDT Plus. 2010; 3(1):8-16.

(3) Canaud B, Morena M, Leray-Moragues H, Chalabi L, Cristol JP. Overview of clinical studies in hemodiafiltration: what do we need now ? Hemodial Int. 2006; 10 Suppl 1:S5-S12.

(4) Canaud B, Morena M, Cristol JP, Krieter D. Beta2-microglobulin, a uremic toxin with a double meaning. Kidney Int. 2006; 69(8):1297-1299.

(5) Cheung AK, Rocco MV, Yan G, Leypoldt JK, Levin NW, Greene T et al. Serum beta-2 microglobulin levels predict mortality in dialysis patients: results of the HEMO study. J Am Soc Nephrol. 2006; 17(2):546-555.

(6) Penne EL, van der Weerd NC, Blankestijn PJ, van den Dorpel MA, Grooteman MP, Nube MJ et al. Role of residual kidney function and convective volume on change in beta2-microglobulin levels in hemodiafiltration patients. Clin J Am Soc Nephrol. 2010; 5(1):80-86.

(7) Penne EL, van der Weerd NC, van den Dorpel MA, Grooteman MP, Levesque R, Nube MJ et al. Short-term effects of online hemodiafiltration on phosphate control: a result from the randomized controlled Convective Transport Study (CONTRAST). Am J Kidney Dis. 2010; 55(1):77-87.

(8) Canaud B, Bragg-Gresham JL, Marshall MR, Desmeules S, Gillespie BW, Depner T et al. Mortality risk for patients receiving hemodiafiltration versus hemodialysis: European results from the DOPPS. Kidney Int. 2006; 69(11):2087-2093.

(9) Donauer J, Schweiger C, Rumberger B, Krumme B, Bohler J. Reduction of hypotensive side effects during online-haemodiafiltration and low temperature haemodialysis. Nephrol Dial Transplant. 2003; 18(8):1616-1622.

(10) Penne EL, van der Weerd NC, Bots ML, van den Dorpel MA, Grooteman MP, Levesque R et al. Patient- and treatment-related determinants of convective volume in post-dilution haemodiafiltration in clinical practice. Nephrol Dial Transplant. 2009; 24(11):3493-3499.

(11) Penne EL, Visser L, van den Dorpel MA, van der Weerd NC, Mazairac AH, van Jaarsveld BC et al. Microbiological quality and quality control of purified water and ultrapure dialysis fluids for online hemodiafiltration in routine clinical practice. Kidney Int. 2009; 76(6):665-672.

(12) Tattersall JE, Ward RA. Online haemodiafiltration: definition, dose quantification and safety revisited. Nephrol Dial Transplant. 2013.

(13) Grooteman MP, van den Dorpel MA, Bots ML, Penne EL, van der Weerd NC, Mazairac AH et al. Effect of online hemodiafiltration on all-cause mortality and cardiovascular outcomes. J Am Soc Nephrol. 2012; 23(6):1087-1096.

(14) Maduell F, Moreso F, Pons M, Ramos R, Mora-Macia J, Carreras J et al. High-Efficiency Postdilution Online Hemodiafiltration Reduces All-Cause Mortality in Hemodialysis Patients. J Am Soc Nephrol. 2013.

(15) Ok E, Asci G, Toz H, Ok ES, Kircelli F, Yilmaz M et al. Mortality and cardiovascular events in online haemodiafiltration (OL-HDF) compared with high-flux dialysis: results from the Turkish OL-HDF Study. Nephrol Dial Transplant. 2012.

(16) Fischbach M, Terzic J, Menouer S, Dheu C, Soskin S, Helmstetter A et al. Intensified and daily hemodialysis in children might improve statural growth. Pediatr Nephrol. 2006; 21(11):1746-1752.

(17) Fischbach M, Terzic J, Menouer S, Dheu C, Seuge L, Zalosczic A. Daily on line haemodiafiltration promotes catch-up growth in children on chronic dialysis. Nephrol Dial Transplant. 2010; 25(3):867-873.

(18) Schaefer F. Daily online haemodiafiltration: the perfect 'stimulus package' to induce growth? Nephrol Dial Transplant. 2010; 25(3):658-660.

(19) Chavers BM, Li S, Collins AJ, Herzog CA. Cardiovascular disease in pediatric chronic dialysis patients. Kidney Int. 2002; 62(2):648-653.

(20) Querfeld U, Anarat A, Bayazit AK, Bakkaloglu AS, Bilginer Y, Caliskan S et al. The Cardiovascular Comorbidity in Children with Chronic Kidney Disease (4C) study: objectives, design, and methodology. Clin J Am Soc Nephrol. 2010; 5(9):1642-1648.

(21) K/DOQI clinical practice guidelines for Nutrition in Children with CKD. Am J Kidney Dis. 2003; 42(4 Suppl 3):S1-201.
