# Supplementary material for: Hemodiafiltration is associated with reduced inflammation, oxidative stress and improved endothelial risk profile compared to high-flux hemodialysis in children
Source: PLoS One. 2018 Jun 18;13(6):e0198320. doi: 10.1371/journal.pone.0198320 (PMC6005477; doi:10.1371/journal.pone.0198320)
Supplement: S1 Table — (PDF) [file pone.0198320.s003.pdf]

S1 Table. Comparison of the biochemistry data of the patients on HD and HDF

	Hemodialysis (n=22)	*Hemodiafiltration (n=22)*	*p value*
Kt/V	1.71±0.28	1.82±0.43	0.30
URR	76.8±6.86	78.4±7.97	0.35
Uric acid, mg/dl	6.27±1.22	5.44±0.74	0.01
Na, mmol/L	138±4.84	140±2.69	0.14
K, mmol/L	4.88±0.9	4.97±0.74	0.79
Ca, mmol/L	2.29±0.19	2.33±0.11	0.36
Phosphorus, mmol/L	1.48±0.38	1.55±0.40	0.50
PTH, pg/ml	186 (75; 437)	131 (42.7; 331)	0.56
Total Cholesterol, mg/dl	139 (131; 176)	146 (117; 184)	0.89
HDL, mg/dl	47.2(34.1; 56.9)	37.0 (32.6; 59.7)	0.39
LDL, mg/dl	74.7(53.2; 94.8)	79.0 (68.2; 107)	0.17
Triglyceride, mg/dl	138 (114; 179)	151 (130; 202)	0.79
Alb, g/L	37.7±3.50	40.0±1.90	0.002
HCO3, mmol/L	22.2±3.86	23.3±3.20	0.32
Hb, g/dl	10.7±1.78	11.3±1.44	0.02
Hct, %	30±5.4	36.6±0.05	<0.001
TSI, %	22.9 (17.7; 32.7)	27.1 (24.4; 35.7)	0.08
Ferritin, ng/ml	282 (135; 543)	304 (217; 612)	0.08

Data was shown as mean±SD; paired sample t-test or median (IQR); Wilcoxon signed-rank test

HD: Hemodialysis, HDF: Hemodiafiltration, URR: Urea reduction rate, PTH: Parathormone, HDL: High density lipoprotein, LDL: Low density lipoprotein, TSI: Transferrin saturation index
