# Supplementary material for: Hemodiafiltration is associated with reduced inflammation, oxidative stress and improved endothelial risk profile compared to high-flux hemodialysis in children
Source: PLoS One. 2018 Jun 18;13(6):e0198320. doi: 10.1371/journal.pone.0198320 (PMC6005477; doi:10.1371/journal.pone.0198320)
Supplement: S2 Table — (PDF) [file pone.0198320.s004.pdf]

**S2 Table**. Comparison of the inflammatory, oxidative stress and endothelial markers between HD and HDF in incident and prevalent patients.

| | *Incident patients (n=13)* | | | | *Prevalent patients (n=9)* | | | | |
|---|---|---|---|---|---|---|---|---|---|
| | *HD* | *HDF* | *$p^1$* | *% change from HD to HDF** | *HD* | *HDF* | *$p^2$* | *% change from HD to HDF** | *$p^3$* |
| Nitrotyrosine, nM/ml | 42.9 (25.1;119) | 36.6 (25.6;42.9) | 0.13 | -32 (-57;66) | 25.7 (23.1;39.0) | 30.4 (22.3;44.5) | 0.78 | 0 (-13;37) | 0.11 |
| Ox-LDL,ng/mL | 248 (198; 334) | 183 (139;214) | 0.01 | -27 (-46;1) | 302 (204;410) | 151 (108;211) | 0.03 | -35 (-66;-11) | 0.42 |
| AGEs, ng/mL | 1334 (1264;1503) | 1029 (962; 1253) | 0.009 | -27 (-36;-3) | 1352 (1088; 1505) | 1120 (989; 1223) | 0.051 | -16 (-30;3) | 0.57 |
| TAC, mmol/L | 0.42 (0.38;0.44) | 1.41 (0.43;2.00) | 0.006 | 264 (13;460) | 0.45 (0.41;1.21) | 2.06 (0.40;3.76) | 0.03 | 82 (-1;394) | 0.07 |
| β2M, mg/L | 37.0 (31.0;43.0) | 22.0 (15.0;25.0) | 0.001 | -43 (-59;-28) | 41.0 (36.5;42.5) | 25.0 (20.0;28.0) | 0.007 | -39 (-51;-23) | 0.33 |
| hsCRP, mg/L | 2.58 (1.76;3.02) | 1.62 (0.46;2.21) | 0.01 | -27 (-75;-5) | 2.86 (2.38;3.35) | 2.21 (1.30;2.95) | 0.08 | -14 (-59;3) | 0.23 |
| IL-6, pg/mL | 3.89 (3.54;11.02) | 4.61 (2.46;14.9) | 0.87 | 3 (-40;35) | 2.68 (1.39;6.64) | 3.19 (2.08;8.59) | 0.21 | 29 (-6;100) | 0.12 |
| IL-10, pg/mL | 8.93 (4.92;17.40) | 6.54 (5.29;11.98) | 0.23 | -26 (-70;72) | 4.18 (1.22;8.61) | 4.43 (2.51;6.35) | 0.85 | 23 (-61;299) | 0.06 |
| ADMA, µmol/L | 0.96 (0.90;1.18) | 0.82 (0.74;1.02) | 0.01 | -21 (-29;5) | 1.12 (0.95;1.26) | 0.86 (0.77;1.03) | 0.02 | -16 (-32;-2) | 0.23 |
| SDMA, µmol/L | 3.11 (2.55; 3.69) | 2.16 (2.08;3.16) | 0.01 | -11 (-43;-4) | 3.05 (2.46;3.55) | 2.82 (2.28;3.14) | 0.19 | -5 (-14;4) | 0.61 |

*Data shown as median (IQR); Wilcoxon signed ranks test*

**% change from HD to HDF was calculated with [((HDF-HD)/HD)*100] formula, No significant difference was found between two groups for %change from HD to HDF.*

*$P^1$Comparison of biomarkers between HD and HDF within incident patients*

*$P^2$ Comparison of biomarkers between HD and HDF within prevalent patients*

*$P^3$Comparison of biomarkers levels in HD, between incident and prevalent patients*

*HD: Hemodialysis, HDF: Hemodiafiltration, Ox-LDL: Oxidized Low density lipoprotein, AGE: Advanced glycation end-products, TAC: Total antioxidant capacity, β2M: Beta 2 microglobuline, hsCRP: High sensitive C-reactive protein, IL: Interleukin, ADMA: Asymmetric dimethylarginine, SDMA: Symmetric dimethylarginine*
