# Supplementary material for: Hemodiafiltration is associated with reduced inflammation, oxidative stress and improved endothelial risk profile compared to high-flux hemodialysis in children
Source: PLoS One. 2018 Jun 18;13(6):e0198320. doi: 10.1371/journal.pone.0198320 (PMC6005477; doi:10.1371/journal.pone.0198320)
Supplement: S3 Table — (PDF) [file pone.0198320.s005.pdf]

S3 Table. Comparison of the inflammatory, oxidative stress and endothelial markers between HD and HDF

	HD	*HDF*	*p value**	*% change from HD to HDF***
Nitrotyrosine, nM/ml	28.5 (23.7; 56)	32.0 (24.4; 43.7)	0.37	-3.50 (-46.6; 53.0)
Ox-LDL,ng/mL	278 (203; 384)	172 (114; 211)	0.001	-27.4 (-56.3; -6.0)
AGEs, ng/mL	1338 (1221; 1490)	982 (1029; 1221)	0.001	-20.1 (-35.1; -0.48)
Total antioxidant capacity, mmol/L	0.43 (0.40; 0.72)	1.68 (0.42; 2.39)	<0.001	165 (2.5; 424)
β2M, mg/L	38.5 (33; 43)	22.5 (16; 26.2)	<0.001	-42.3 (-54.1; -26.7)
hsCRP, mg/L	2.80 (1.95; 3.16)	1.92 (0.70; 2.43)	0.002	-20.3 (-68.2; -1.34)
IL-6, pg/mL	3.72 (2.34; 8.36)	3.76 (2.37; 8.86)	0.50	20.1 (-26.9; 63.8)
IL-10, pg/mL	6.23 (3.16; 10.5)	5.73 (4.39; 10.4)	0.40	-6.00 (-62.0; 63.0)
ADMA, µmol/L	1.03 (0.92; 1.21)	0.85 (0.75; 1.02)	0.001	-19.0 (-29.8; 0.40)
SDMA, µmol/L	3.54 (2.46; 3.54)	2.58 (2.12; 3.12)	0.003	-11.4 (-23.3; 1.62)

** Data shown as median (IQR); Wilcoxon signed ranks test*
*** % change from HD to HDF was calculated with [((HDF-HD)/HD)*100] formula*
HD: Hemodialysis, HDF: Hemodiafiltration, Ox-LDL: Oxidized Low density lipoprotein, AGE: Advanced glycation end-products, β2M: Beta 2 microglobuline, hsCRP: High sensitive C-reactive protein, IL: Interleukin, ADMA: Asymmetric dimethylarginine, SDMA: Symmetric dimethylarginine
